# Otorhinolaryngological manifestations of coronavirus disease 2019: a prospective review of 600 patients

**DOI:** 10.1017/S0022215121000220

**Published:** 2021-01-18

**Authors:** S Bhatta, S Gandhi, S J Saindani, D Ganesuni, A D Ghanpur

**Affiliations:** Department of Laryngology (ENT), Deenanath Mangeshkar Hospital and Research Centre, Pune, India

**Keywords:** COVID-19, Prospective Studies, Comorbidity

## Abstract

**Objectives:**

To evaluate otorhinolaryngological manifestations of coronavirus disease 2019 infection and the time required for their resolution.

**Methods:**

A prospective analysis was conducted of coronavirus disease 2019 patients presenting from 1 April 2020 to 30 July 2020. The otorhinolaryngological manifestations were evaluated based on patient history. The time required for symptom resolution was evaluated separately for intensive care unit and non-intensive care unit patients.

**Results:**

A total of 600 patients were included in the study; 13.3 per cent required the intensive care unit and 2.2 per cent expired. The otorhinolaryngological manifestations were: sore throat (88 per cent), fever (78.8 per cent), anosmia or hyposmia (63.6 per cent), ageusia or hypogeusia (63.5 per cent), rhinorrhoea (51.3 per cent), nasal obstruction (33.5 per cent), sneezing (30.3 per cent), and breathing difficulty (18.6 per cent). The time required for symptom resolution was longest for breathing difficulty (23.6 days for intensive care unit and 8.2 days for non-intensive care unit patients).

**Conclusion:**

Otorhinolaryngological symptoms are one of the main presentations of coronavirus disease 2019 infection. The increased prevalence of medical co-morbidities in patients requiring intensive care unit and in deceased patients is also highlighted.

## Introduction

The infection caused by a novel coronavirus (severe acute respiratory syndrome coronavirus-2 (SARS-CoV-2)), the coronavirus disease 2019 (Covid-19), has spread throughout the world. It was declared a pandemic by the World Health Organization on 11 March 2020.^1^ Coronavirus disease 2019 primarily affects the respiratory system, although it can involve other organ systems of the body.^2^ The nasal and the oral cavity are the main portal of entry of the virus, with the nasal cavity and the nasopharynx regarded as the most common sites for virus replication.^3^

The Covid-19 infection presents with various manifestations, such as fever, cough, sore throat, breathing difficulty, headache, olfactory and gustatory symptoms, diarrhoea, myalgia, nasal obstruction, rhinorrhoea, fatigue, expectoration, and post-nasal drip.^4–11^ There have been no recognised studies in the literature on pathognomonic signs and symptoms for the diagnosis of the Covid-19 infection. The presentation of Covid-19 infection is becoming more and more diverse as the transmission crosses geographical borders.

This study describes the otorhinolaryngological manifestations of Covid-19 infection, with an intention to help physicians diagnose the Covid-19 infection at the earliest stage. We found only one study in the literature, a review article, that described the ENT manifestations of Covid-19.^7^ There are studies, however, that have described loss of smell and taste as a manifestation of Covid-19 infection.^9,10,12–18^ All these studies were performed in either Chinese or European populations. Studies presenting such data from Indian subcontinent patients are lacking. This study provides a platform to understand the Covid-19 disease and its manifestations in the Indian subcontinent, to better understand the epidemiology for this part of the world.

## Materials and methods

This prospective study was performed to evaluate the otorhinolaryngological manifestations of Covid-19 positive patients presenting from 1 April 2020 to 30 July 2020. The primary objectives of the study were to identify the otorhinolaryngology-related signs and symptoms in patients with Covid-19 infection and to determine the time required for symptom resolution.

The study was carried out at the ENT Department, Deenanath Mangeshkar Hospital and Research Center, Pune, India. Approval for the study was granted by the hospital's institutional ethics committee. Consent for study involvement was obtained from every patient included in the study. The patients were assured about the confidentiality of the details they provided.

All patients visiting the hospital, irrespective of the presenting complaint, were screened for Covid-19 related symptoms. Those patients suspicious for Covid-19 infection were referred to the Covid-19 clinic. In the Covid-19 clinic, patients were further evaluated and, if needed, scheduled for a reverse transcriptase polymerase chain reaction test, at the physician's discretion. Patients who had been in contact with a known Covid-19 patient, and those with recent international travel history or who had travelled to Covid-19 high-risk zones, were also subjected to reverse transcriptase polymerase chain reaction testing. The hospital protocol required the admission of all patients who tested positive for Covid-19 on reverse transcriptase polymerase chain reaction testing. In the admitted Covid-19 positive patients, the reverse transcriptase polymerase chain reaction test was performed every 3rd day. The Covid-19 positive patients were discharged only after the reverse transcriptase polymerase chain reaction test result was negative for Covid-19.

This study included the patients visiting the hospital, of all ages and genders, who tested positive for Covid-19 on reverse transcriptase polymerase chain reaction testing. The demographic details and a detailed history were recorded for all Covid-19 positive patients. The patients were asked specifically about any ENT symptoms. The presenting otorhinolaryngology-related complaints were recorded as separate symptoms. The time since symptom onset was also calculated.

The numbers of patients managed in the intensive care unit and the non-intensive care unit wards were recorded separately. For the admitted Covid-19 positive patients, the morning rounds were performed every day to inquire about the status of the symptoms. The time taken for symptom resolution was documented, from the time of admission to the time of resolution. The data for symptom resolution duration were recorded separately for patients managed in the intensive care unit and the non-intensive care unit wards. The presence of co-morbidities, or lack thereof, was evaluated and documented for every Covid-19 positive patient.

## Results

A total of 600 patients (337 (56.2 per cent) males and 263 (43.8 per cent) females) were included in the study. The average age of the patients was 42.9 ± 12.6 years (range, 18–78 years). Of the 600 patients, 80 (13.3 per cent) were managed in the intensive care unit and the remaining 520 (86.7 per cent) were managed in the isolation ward. The average age of patients admitted into the intensive care unit was 69.3 ± 18.4 years. All the patients admitted to the intensive care unit had breathing difficulty at presentation and had at least one medical co-morbidity. Of the 80 intensive care unit patients, 13 (16.2 per cent) expired. Each one of these 13 deceased patients had at least two medical co-morbidities. The average age of the deceased patients was 73.2 ± 2.3 years.

**Fig. 1.**
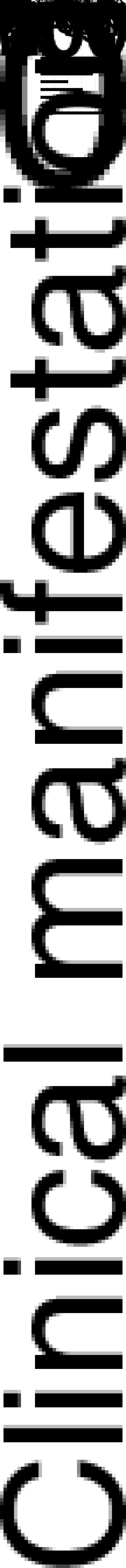
Distribution of patients based on clinical manifestations.

The distribution of patients on the basis of clinical manifestations is shown in Figure 1. The clinical manifestations were primarily classified as otorhinolaryngology-related symptoms (fever, sore throat, runny nose, nasal obstruction, anosmia or hyposmia, ageusia or hypogeusia, sneezing, and breathing difficulty) and other symptoms (cough, headache, myalgia, fatigue, vomiting and diarrhoea). There were no patients with any significant ear-related complaints. Nasal obstruction, affecting 201 patients, was unilateral at presentation in 31 (15.4 per cent) and bilateral in 170 (84.4 per cent) patients. Seventy-two of the 600 patients (12 per cent) had no symptoms. Of these asymptomatic patients, 48 out of 600 (8 per cent) had a history of exposure to Covid-19 patients, and 24 (4 per cent) had a history of international travel or travel to a high-risk zone.

**Fig. 2.**
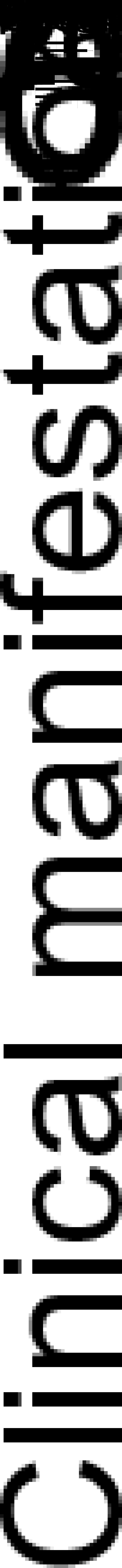
Time since symptom onset (with respective standard deviations).

The time since symptom onset, for the various clinical manifestations, is shown in Figure 2. The patients with anosmia or hyposmia had the longest symptom duration, while patients with breathing difficulty had the shortest symptom duration.

**Fig. 3.**
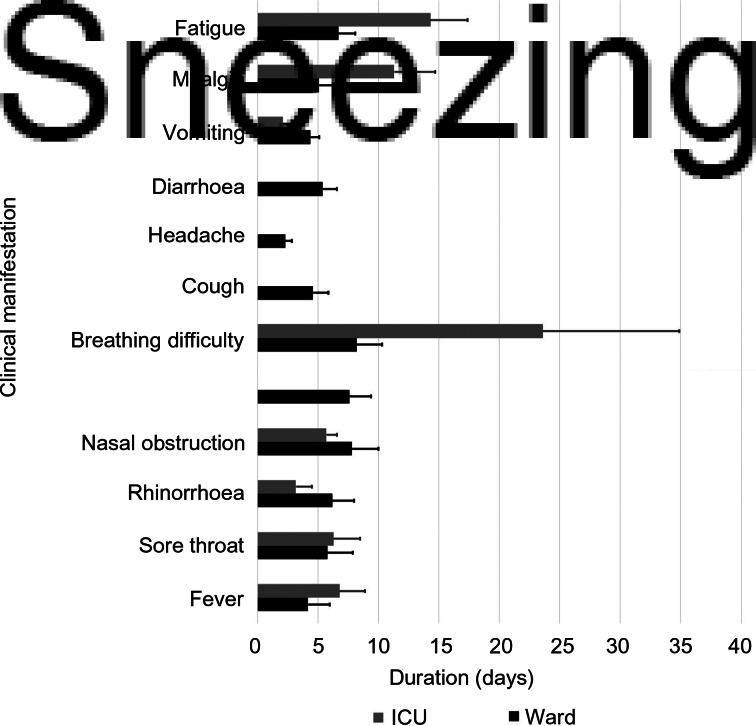
Time needed for symptom resolution (along with standard deviations), both for intensive care unit (ICU) and non-intensive care unit patients.

The time needed for the resolution of a particular symptom was calculated (Figure 3). Figure 3 shows the symptoms that had resolved by the time the patient was discharged from the hospital, after testing negative for Covid-19 on reverse transcriptase polymerase chain reaction testing. The anosmia or hyposmia, and ageusia or hypogeusia, were not resolved in 335 of 382 patients (87.7 per cent) and 353 out of 381 patients (92.6 per cent) at the time of discharge, respectively. These patients are not included in Figure 3, nor are the 13 deceased patients. The time needed for symptom resolution was recorded separately for the intensive care unit and the non-intensive care unit patients.

**Fig. 4.**
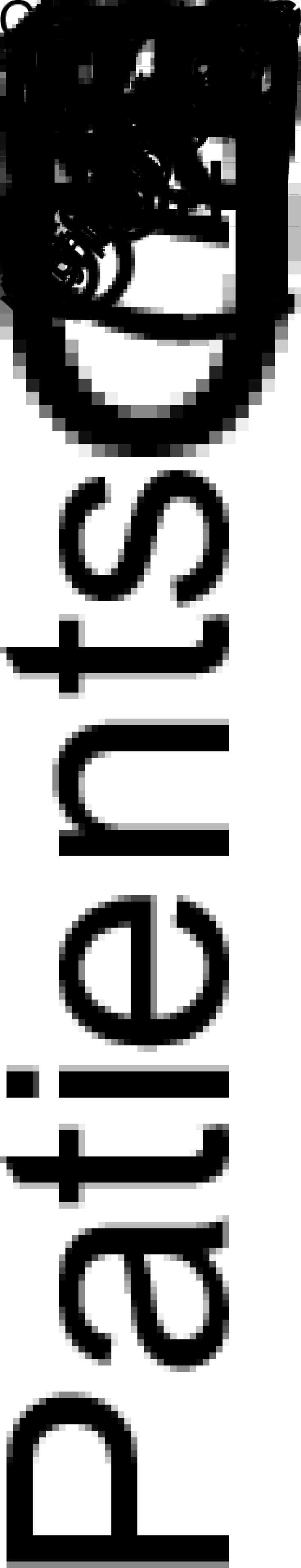
Distribution of patients according to medical co-morbidities. COPD = chronic obstructive pulmonary disease; CVD = cardiovascular disease

The study showed that medical co-morbidities were present in 228 patients (38 per cent), as shown in Figure 4. Of the 600 patients, 138 (23 per cent), 68 (11.3 per cent) and 22 (3.6 per cent) had one, two, and more than two co-morbidities, respectively.

## Discussion

The manifestations of Covid-19 infection vary greatly;^6,9,19–21^ patients may present without any symptoms, or may have symptoms suggestive of shock with multi-organ failure.^7^ This study has shown the diversity in the presentation of patients with Covid-19 infection relating to otorhinolaryngology. The demographic make-up of the patients is comparable with that reported in other similar studies.^4,5,7–9^ The hospital-maintained protocol was to admit all patients positive for Covid-19 on reverse transcriptase polymerase chain reaction testing, so they could be isolated in order to control the spread of the infection to the community.

Intensive care unit management was required for 13.3 per cent of the patients in this study. Chen *et al*.^22^ and Lodigiani *et al*.^23^ showed that intensive care unit admission was required for 23 per cent and 16 per cent of the total Covid-19 patients in their studies, respectively. The case fatality rates reported by Onder *et al*.^24^ were 7.2 per cent in Italy and 2.3 per cent in China. A study by Russell *et al*.^25^ showed the case fatality rate to be 2.6 per cent on board the Diamond Princess cruise ship. The case fatality rate reported in our study was 2.2 per cent. In our study, the patients who died had at least two co-morbidities, and their average age was 73.2 ± 2.3 years, similar to other studies surveyed.^6,24–26^

This study primarily focused on otorhinolaryngological manifestations of Covid-19 infection; hence, the presenting signs and symptoms in patients were divided into otorhinolaryngology-related and other manifestations. The otorhinolaryngological symptoms in this study, in decreasing order of frequency, were: sore throat (88 per cent), fever (78.8 per cent), anosmia or hyposmia (63.6 per cent), ageusia or hypogeusia (63.5 per cent), rhinorrhoea (51.3 per cent), nasal obstruction (33.5 per cent), sneezing (30.3 per cent), and difficulty breathing (18.6 per cent). Fever was considered an otorhinolaryngological symptom as it is systemic and non-specific, and its presence can be attributed to an inflammatory response in the upper airway resulting from Covid-19 infection. Difficulty breathing was considered an otorhinolaryngological symptom for similar reasons. A large number of patients with Covid-19 infection have visited the otorhinolaryngology clinic with breathing difficulty, indicating an issue within the upper airway.

No patients had any ear-related complaint in this study. Mustafa^27^ evaluated the audiological profile in Covid-19 positive patients and demonstrated significantly increased hearing loss among the patients with Covid-19 infection. However, no other study has so far demonstrated any significant impact of Covid-19 on hearing mechanisms. Hence, routine audiometry was not performed for Covid-19 patients in this study, to avoid the unnecessary exposure of healthcare professionals.

The other manifestations observed in this study were: cough (87 per cent), headache (82 per cent), myalgia (67 per cent), fatigue (63.5 per cent), diarrhoea (14.3 per cent) and vomiting (13 per cent). The clinical manifestations in Covid-19 patients surveyed from various other studies in the literature are shown in Table 1.^4,6,7,12,14,19,20,22,28^

**Table 1.** Clinical manifestations of Covid-19 infection as shown by different studies

Manifestation	Lechien *et al*.^12^	Huang *et al*.^4^	Guan *et al*.^20^	Wang *et al*.^6^	Chen *et al*.^22^	Speth *et al*.^14^	Li *et al*.^28^	El-Anwar *et al*.^7^	Liu *et al*.^19^
Fever	48	94.1	87.9	98.6	83	74.8	88.5	73.5	81.8
Cough	78	50	67.7	59.4	82	68	68.6	61.1	48.2
Sore throat	–	–	13.9	–	5	–	–	11.3	–
Runny nose	–	–	–	–	4	–	–	2	–
Nasal obstruction	–	–	4.8	–	–	49.5	–	3.4	–
Ageusia or hypogeusia	88	–	–	–	–	65	–	–	–
Anosmia or hyposmia	85.6	–	–	–	–	61.2	–	–	–
Sneezing	–	–	–	–	–	–	–	–	–
Breathing difficulty	–	14.7	18.6	3.2	31	46.6	21.9	16.2	19
Headache	46	5.9	13.6	6.5	8	–	12.1	10.7	9.5
Myalgia	–	23.5	13.6	34.8	11	–	35.8	10.4	32.1
Vomiting	23	–	5	3.6	1	–	3.9	3.9	–
Diarrhoea	51	14.7	3.7	10.1	2	–	4.8	4.2	8
Loss of appetite	52	–	–	39.9	–	–	–	–	–
Fatigue	–	–	69.6	69.6	–	–	–	11.2	–
Expectoration	–	–	33.4	–	–	–	28.2	–	4.4
Chest pain	–	–	–	–	2	–	–	0.1	–

All data represent percentages of patients. Covid-19 = coronavirus disease 2019

In this study, 12 per cent of patients with Covid-19 infection were asymptomatic. Mizumoto *et al*.,^29^ in their study of individuals on board the Diamond Princess cruise ship, and He *et al*.,^30^ in their systematic review and meta-analysis, found the rate of asymptomatic cases to be 17.9 per cent and 15.6 per cent of total positive cases, respectively.

The time required for symptom resolution was shortest for breathing difficulty (1.3 ± 0.2 days), followed by fever (2.4 ± 0.9 days). These were the alarming symptoms that brought the patients to the hospital the earliest. The increased time needed for symptom resolution for anosmia or hyposmia (14.3 ± 2.8 days), ageusia or hypogeusia (13.2 ± 1.8 days), nasal obstruction (10.1 ± 3.1 days), sneezing (8.3 ± 1.3 days), sore throat (7.6 ± 2.3 days), and rhinorrhoea (6.3 ± 1.2 days) indicates a decreased awareness regarding these symptoms being manifestations of Covid-19 infection.

The time required for symptom resolution in this study was calculated separately for patients managed in the intensive care unit and the ward (Figure 3). It was found that breathing difficulty, fatigue, myalgia, fever and sore throat persisted for a longer time in the intensive care unit patients compared to the non-intensive care unit patients. To our knowledge, no previous study has evaluated symptom resolution in this manner.

A study by Lechian *et al*.^12^ showed that olfactory symptoms were recovered in 33 per cent, 39.6 per cent, 24.2 per cent and 3.3 per cent of patients in 1–4 days, 5–8 days, 9–14 days and more than 15 days, respectively. Similarly, they also showed that gustatory symptoms were relieved in 20.3 per cent, 47.5 per cent, 28.8 per cent and 3.4 per cent of patients in 1–4 days, 5–8 days, 9–14 days and more than 15 days, respectively. This study demonstrated rather contrasting findings, as 87.7 per cent of olfactory symptoms and 92.6 per cent of gustatory symptoms had not resolved by the time the reverse transcriptase polymerase chain reaction test was negative for Covid-19. This study did not use an objective assessment for evaluating olfactory and gustatory symptoms, as was employed by Vaira *et al*.^10^

Medical co-morbidities were present in 38 per cent of patients in this study, with diabetes in 19.6 per cent, hypertension in 9.2 per cent, chronic obstructive pulmonary disease in 8 per cent, neurological disease in 6.8 per cent, asthma in 6.3 per cent, allergic rhinitis in 4.3 per cent, renal disease in 4.3 per cent and cardiovascular disease in 3.8 per cent of the patients studied. Medical co-morbidities were present in all patients who required intensive care unit support. The medical co-morbidities of Covid-19 patients reported in various other studies are shown in Table 2.^4,6,20,22,31^ The presence of medical co-morbidities was associated with a poorer prognosis in patients with Covid-19 infection, as shown by the current study and other investigations.^2,21,24,25,31,32^

- Prospective analysis was conducted of ENT manifestations and their duration in 600 coronavirus disease 2019 positive patients
- Intensive care unit management was required in 13.3 per cent of patients, who had at least one medical co-morbidity
- Of patients, 2.2 per cent expired; these individuals had at least two medical co-morbidities
- ENT manifestations included: sore throat, fever, anosmia or hyposmia, ageusia or hypogeusia, rhinorrhoea, nasal obstruction, sneezing, and difficulty breathing
- Patients with breathing difficulty and fever presented earlier than those with other symptoms
- Of the symptoms, breathing difficulty required the longest time for resolution

**Table 2.** Medical co-morbidities of Covid-19 patients as demonstrated by various studies

Co-morbidity	Huang *et al*.^4^	Wang *et al*.^6^	Chen *et al*.^22^	Guan *et al*.^20^	Singh *et al*.^31^
Total	47.1	46.4	–	25.1	34.3
Diabetes	11.8	10.1	13	8.2	10.5
Hypertension	23.5	31.2	–	16.9	20.7
COPD	2.9	2.9	1	1.5	2
Neurological disease	–	5.1	1	–	–
Renal disease	–	2.9	–	1.3	1.2
Cardiovascular disease	17.6	–	40	3.7	7.4
Allergic rhinitis	–	–	–	–	–
Malignancy	8.8	7.2	1	1.1	–
AIDS	5.9	1.4	–	0.2	–
Chronic liver disease	2.9	2.9	11	1.8	3.2

All data represent percentages of patients. Covid-19 = coronavirus disease 2019; COPD = chronic obstructive pulmonary disease; AIDS = acquired immune-deficiency syndrome

The primary limitations of the study were felt to be the investigation of Covid-19 cases from a single institute, the limited study period and the lack of follow-up evaluation. The number of cases, taken from a single institute, limits knowledge of geographical and institutional variations regarding the presentation of Covid-19 infection. The lack of patient follow-up evaluation after discharge hinders the detection of any re-emergence of symptoms or re-infection from Covid-19. This study suggests a need for further large-scale research, with the involvement of multiple institutes from varied geographical locations, and with a greater sample size, to enable better understanding of Covid-19 symptomatology.

## Conclusion

Otorhinolaryngological symptoms are one of the main presentations of Covid-19 infection, with sore throat and fever being the most common. The increased prevalence of medical co-morbidities in patients requiring intensive care unit management and in deceased patients has also been highlighted. The lack of data variation associated with the involvement of a single institute, and the lack of follow-up evaluation, were felt to be the primary shortcomings of the study. We therefore call for a larger, multi-centre study, with follow-up evaluation.
